# Tree Root Zone Microbiome: Exploring the Magnitude of Environmental Conditions and Host Tree Impact

**DOI:** 10.3389/fmicb.2020.00749

**Published:** 2020-04-23

**Authors:** Jean de Dieu Habiyaremye, Kezia Goldmann, Thomas Reitz, Sylvie Herrmann, François Buscot

**Affiliations:** ^1^Department of Soil Ecology, Helmholtz Centre for Environmental Research (UFZ), Halle, Germany; ^2^Department of Biology II, Leipzig University, Leipzig, Germany; ^3^Department of Mathematics, Science and Physical Education, University of Rwanda, Kigali, Rwanda; ^4^German Centre for Integrative Biodiversity Research (iDiv) Halle-Jena-Leipzig, Leipzig, Germany

**Keywords:** PhytOakmeter, microbial recruitment, microbial diversity, environmental conditions, core and site-specific microbiomes

## Abstract

Tree roots attract their associated microbial partners from the local soil community. Accordingly, tree root-associated microbial communities are shaped by both the host tree and local environmental variables. To rationally compare the magnitude of environmental conditions and host tree impact, the “PhytOakmeter” project planted clonal oak saplings (*Quercus robur* L., clone DF159) as phytometers into different field sites that are within a close geographic space across the Central German lowland region. The PhytOakmeters were produced via micro-propagation to maintain their genetic identity. The current study analyzed the microbial communities in the PhytOakmeter root zone vs. the tree root-free zone of soil two years after out-planting the trees. Soil DNA was extracted, 16S and ITS2 genes were respectively amplified for bacteria and fungi, and sequenced using Illumina MiSeq technology. The obtained microbial communities were analyzed in relation to soil chemistry and weather data as environmental conditions, and the host tree growth. Although microbial diversity in soils of the tree root zone was similar among the field sites, the community structure was site-specific. Likewise, within respective sites, the microbial diversity between PhytOakmeter root and root-free zones was comparable. The number of microbial species exclusive to either zone, however, was higher in the host tree root zone than in the tree root-free zone. PhytOakmeter “core” and “site-specific” microbiomes were identified and attributed to the host tree selection effect and/or to the ambient conditions of the sites, respectively. The identified PhytOakmeter root zone-associated microbiome predominantly included ectomycorrhizal fungi, yeasts and saprotrophs. Soil pH, soil organic matter, and soil temperature were significantly correlated with the microbial diversity and/or community structure. Although the host tree contributed to shape the soil microbial communities, its effect was surpassed by the impact of environmental factors. The current study helps to understand site-specific microbe recruitment processes by young host trees.

## Introduction

The soil microbiome, the community of soil microorganisms and their genomes (Scher and Abramson, 2011), steers many ecological processes in soils and determines plant health (Aislabie et al., 2013) and productivity (Berg, 2009). Impacts of soil microorganisms on plants include increased nutrient availability and uptake (Lugtenberg et al., 2002; Morrissey et al., 2004), disease suppression (Mendes et al., 2011), as well as increased tolerance against abiotic (Zolla et al., 2013) and biotic stressors (Zamioudis and Pieterse, 2011). Microorganisms have abilities to rapidly adapt to changing environmental conditions (Gehring et al., 2017; Lau et al., 2017). Therefore, the “plant root microbiome” can be considered as “the powerhouse of plant adjustment to local conditions” (Vandenkoornhuyse et al., 2015).

The “plant root microbiome” originates from the local soil microbial community, and is shaped by the root exudate composition (Bais et al., 2006; Lareen et al., 2016). On the one hand, the composition of plant root-associated microbial communities across various ecosystems has been reported to highly depend on environmental parameters (Bulgarelli et al., 2012; Lundberg et al., 2012) such as climate and weather (Brockett et al., 2012; Lladó et al., 2018), but also on soil chemistry, especially pH and organic matter content (Zhou et al., 2002; Rousk et al., 2010; Lareen et al., 2016). However, in soils with similar edaphic parameters and climatic conditions, there can be significant local heterogeneity in the composition of soil bacterial and fungal communities even within the same region (Bokulich et al., 2014; Gourmelon et al., 2016). This may partly result from variations of unmeasured environmental parameters across the sampled field sites (Landesman et al., 2014) or from dispersal limitation among members of the microbial community (Bissett et al., 2010). On the other hand, the constituents of plant root exudates (sugars, vitamins, nucleotides, flavones, auxins, and stimulators), which differ between plant species and even among plant genotypes within a species (Broeckling et al., 2008), are also considered as important drivers structuring soil microbial communities proliferating in the plant root zone (Dotaniya and Meena, 2015). However, separating the effects of heterogeneity in environmental conditions within a region from those induced by variability of exudates between plant individuals is largely unexplored.

Oak, a foundation tree species, displays among the highest levels of below and aboveground biotic interactions in European forests (Plomion et al., 2018). More than 20 years ago, numerous investigations have been made on how oak trees harmonize their own development, biotic interaction and adaptation to the environment. These studies were through microcosm experiments using micro-cuttings of the oak clone DF159 (*Quercus robur* L.) with different analytic approaches including transcriptomics (Herrmann et al., 1998, 2015, 2016; Tarkka et al., 2013). More recently, clonal saplings regenerated from DF159 were planted in TERENO^1^ field sites as “phytometers” (Herrmann et al., 2016; Ferlian et al., 2018), i.e., standardized plants transplanted into different environments to serve as environmental measuring “instruments” (Dietrich, 2013). The tree phytometer system using clone DF159 is called “PhytOakmeter” (Ferlian et al., 2018). A few years after outplant in the field, the PhytOakmeter saplings have been shown to exert an impact on the biological activity in their surrounding soil (Eisenhauer et al., 2018). Therefore, PhytOakmeter has the potential to help unraveling the tree-related factors that shape the root microbiome.

Previous investigations on soil microorganisms associated with plant roots focused on rhizospheric soil microbial communities (Grayston et al., 1998; Fang et al., 2001; Nunan et al., 2005; Hartmann et al., 2009; Haldar and Sengupta, 2015). However, as a shared environment between plant roots and microbes (Jacoby et al., 2017), the rhizosphere is most directly controlled by the selective forces exerted by host plants (Kowalchuk et al., 2002). Some studies reported an enhanced microbial species richness and diversity in the rhizosphere due to its enrichment in resources (Novello et al., 2017). However, there is an opposite view that, due to selection property of root exudates, the rhizosphere may comprise a strongly reduced proportion of the soil microorganisms (Philippot et al., 2013). In any case, rhizosphere-focused studies do not give enough weight to the contribution of environmental factors in shaping the microbiome of the root zone of soil. Therefore, investigating soil of the root zone by discarding the rhizosphere soil *senso stricto* enables to rationally analyze the respective impacts of plant and environment factors in shaping the plant root microbiome (Weißbecker et al., 2018).

Using PhytOakmeters planted in plots within the same central German region and under comparable climate conditions, the current study aimed to distinguish between the impacts of tree-mediated recruitment and local environmental factors on microbial diversity and community structure by comparing the tree root zone vs. the tree root-free zone of the soils. The study was performed using Illumina pair-end amplicon sequencing targeting the small subunit (SSU) of the 16S and the internal transcribed spacer (ITS) region of the 18S rDNA to gain bacteria and fungi, respectively. As result of the common genetic identity of the clonal saplings and of the homogeneity in climate conditions, we hypothesized a high similarity in microbial diversity and community structure within root zones of PhytOakmeters planted in Central German TERENO grassland field sites. Due to an extended rhizosphere mediated selection effect of the host tree, we expected a lower microbial diversity in the PhytOakmeter root zone than in the tree root-free zone within respective field sites. In comparison to the tree root-free zone, we expected to find higher abundance of some particular soil microbial taxa, due to creation in the PhytOakmeter root zone of a particular niche which selects specific microbial taxa.

## Materials and Methods

### Field Sites and PhytOakmeter

The PhytOakmeter experiment was carried out in central Germany at four TERENO grassland field sites: Harsleben (51°51′43.43″ N, 11°04′58.73″ W, 138 m), Pfeiffhausen (51°37′47.68″ N, 11°42′19.95″ W, 137 m), Greifenhagen (51°37′20.80″ N, 11°24′59.62″ W, 292 m) and Bad Lauchstädt (51°23′29.65″ N, 11°52′32.14″ W, 119 m). Because of their geographic proximity, the PhytOakmeter field sites share comparable weather conditions (Supplementary Table S1). Due to the continental climate, flatness and position in the rain shadow of the Harz Mountains, this region is warm and dry with annual precipitations usually less than 500 mm (Wollschläger et al., 2016).

The DF159 oak tree saplings were produced via micropropagation which warrants their common genetic identity (Herrmann et al., 2016), and in November 2014, 2-year PhytOakmeter trees were outplanted in grassland sites. The distance between trees ranges from 6 to 10 m according to individual field plots. Beside the oaks, the entire soil surface of all field sites was covered by herbaceous plants as illustrated by Harsleben field site in Supplementary Figure S1. In September 2016, six core trees per site were randomly selected for this study. To determine tree performance and, later on, correlate it with soil microbial community structure, tree height at outplanting as well as tree percentage height increases in 2015 and 2016 were measured using a meter ruler. Moreover, number of shoot flushes produced by main stems of the core trees during the 2016 vegetation period were counted, and, as a proxy reflecting biomass production in each flush, five leaves were taken from every shoot flush of each tree. As core trees of all the sites had grown at least one shoot flush (SF1), we only considered the leaf biomass of the first shoot flushes during subsequent analyses.

### Soil Sampling

In total, 38 soil samples were taken in September 2016: 24 samples in the tree root zone (6 trees per site × 4 sites = 24 soil samples) and 14 samples in the tree root-free zone that were used to analyze local soil microbial pools (4 samples per site in Harsleben and Pfeiffhausen, 3 samples per site in Greifenhagen and Bad Lauchstädt). At each field site, PhytOakmeter root zone and the tree root-free zone soil samples were taken within the same plot. Each soil sample consisted of three subsamples which were mixed to constitute a composite sample as illustrated by Harsleben plot sampling design in Supplementary Figure S2. All samples were collected using a 2 cm diameter soil auger to a 10 cm soil depth.

The soil samples were sieved using 2 mm mesh size to remove debris and homogenize the soil sample before being packed into sampling bags. From each sieved sample, one aliquot (±50 g) was kept for soil chemical analyses and another aliquot (±10 g) for molecular analyses, and both were stored at -20°C directly after sampling.

### Soil Chemical Analysis

Sixteen soil chemical parameters were analyzed (Table 1). Soil pH was determined with a glass electrode after 1 h in a suspension 1:2.5 mixture of soil and 0.01 M CaCl_2_ as in Moche et al. (2015) and Goldmann et al. (2015). Total soil carbon (TC) and nitrogen (TN) were determined in triplicate by dry combustion using a Vario EL III C/H/N analyzer (Elementar, Hanau, Germany). Due to negligible carbonate concentration of the soil samples (<2%), the obtained total carbon was taken to represent soil organic carbon, SOC (Francioli et al., 2016). To have an idea on the content of soluble soil organic matter, hot water extractable C (HWC) was measured as in Francioli et al. (2016) and N (HWN) as in Schulz et al. (2011). Cold water extraction of organic matter content was performed to measure the amount of labile and easily available organic carbon and nitrogen, representing the nutritional pool for these elements at the sampling time (Zsolnay, 1996). Cold water extractable carbon (CWC) and nitrogen (CWN) were then determined as in Schmidt et al. (2017). Mineral nitrogen contents (NH_4_^+^-N and NO_3_^–^-N) were measured as in Francioli et al. (2016). Available P and K were extracted from soil with calcium acetate lactate (1:20 w/v, pH 4.2, 1.5 h) (Schüller, 1969). After filtration of the suspension (filter type: Whatman Schleicher and Schuell 595 1/5 Ø 270 mm), P and K were quantified in 1:10 diluted extracts by inductively coupled plasma optical emission at emission lines 766.49 nm (K) and 178.287 nm (P) using a SPECTRO ARCOS spectrometer (Spectro Analytical Instruments GmbH, Kleve, Germany).

**TABLE 1:** Chemical parameters of the soil samples: pH, soil organic carbon (SOC), total soil nitrogen (TN), carbon-to-nitrogen ratio (C/N), Cold water extractable carbon (CWC) and nitrogen (CWN), CWC-to-CWN ratio (CWC/CWN), hot water extractable carbon (HWC) and N (HWN), HWC-to-HWN ratio (HWC/HWN), soil moisture, ammonium and nitrate-bound nitrogen (NH_4_^+^-N and NO_3_^–^-N), total mineral nitrogen (min.N), potassium (K), and phosphorous (P).

Parameter	Harsleben	Pfeiffhausen	Greifenhagen	Bad Lauchstädt
pH	7.6(±0.3)^a^	7.5(±0.4)^a^	7.5(±0.5)^a^	6.3(±0.2)^b^
SOC (%)	2.6(±0.4)^a^	2.9(±0.4)^a^	1.3(±0.2)^c^	2.1(±0.1)^b^
TN (%)	0.15(±0.03)^b^	0.18(±0.01)^a^	0.11(±0.03)^c^	0.14(±0.01)^b^
C/N	18.0(±4.5)^a^	15.9(±1.2)^a^	12.0(±4.2)^b^	14.9(±0.4)^ab^
CWC (mg/kg)	79.9(±14.8)^b^	96.8(±13.7)^a^	58.4(±10.6)^c^	97.7(±14.4)^a^
CWN (mg/kg)	5.3(±0.9)^c^	7.7(±1.1)^a^	5.7(±1.1)^b^	7.6(±1.4)^a^
CWC/CWN	15.4(±3.3)^a^	12.6(±1.2)^b^	10.5(±2.8)^c^	13.3(±3.2)^abc^
HWC (mg/kg)	1065.7(±166.6)^b^	1437.0(±164.3)^a^	627.8(±139.4)^c^	616.8(±61.3)^c^
HWN (mg/kg)	101.7(±19.9)^b^	142.5(±18.3)^a^	62.7(±14.7)^c^	60.8(±7.3)^c^
HWC/HWN	10.5(±0.6)	10.1(±0.6)	10.1(±0.4)	10.2(±0.8)
Soil moisture (%)	6.9(±1.1)^a^	5.5(±1.4)^b^	7.5(0.7)^a^	7.6(0.6)^a^
NH_4_^+^-N (mg/kg)	3.2(±0.5)^ab^	3.7(±0.7)^a^	2.5(±0.9)^b^	2.6(±1.1)^b^
NO_3_^–^-N (mg/kg)	1.0(±0.8)	1.0(±0.5)	0.5(±0.4)	0.9(±1.4)
min.N (mg/kg)	4.2(±1.2)^a^	4.6(±1.1)^a^	3.1(±1.1)^b^	3.1(±2.1)^ab^
K (mg/kg)	156.1(±87.0)	153.9(±39.9)	199.3(±82.4)	148.2(±52.3)
P (mg/kg)	54.5(±47.3)^ab^	51.8(±9.9)^a^	33.1(±21.5)^b^	24.0(±6.9)^b^

*Values represent means (± standard deviation). Different superscript letters after standard deviations in a row mean statistically different (p < 0.05) according a one-way ANOVA and Tukeys’ HSD test.*

### DNA Extraction, Amplification, and Sequencing

Total microbial DNA was extracted from 0.4 g of each soil composite sample using the Power Soil DNA Isolation Kit (Qiagen, Hilden, Germany), following the manufacturer’s instructions. The concentrations of DNA extracts were determined with a NanoDrop-8000 spectrophotometer (Thermo Fisher Scientific, Dreieich, Germany). DNA extracts were stored at −20°C, and adjusted to 10–15 ng/μl prior to PCR amplification. PCR genomic DNA amplicon libraries of the targeted microorganisms (bacteria and fungi) were produced from the genomic DNA templates. The bacterial 16S and fungal ITS2 within the rDNA region were amplified using a modified primer mix: P5_8N_515F + P5_7N_515F (forward) together with P7_2N_806R + P7_1N_806R (Caporaso et al., 2012; Moll et al., 2018) for the bacteria, and P5-5N-ITS4 (Gardes and Bruns, 1993; Leonhardt et al., 2019)/P7-4N-fITS7 (Ihrmark et al., 2012; Leonhardt et al., 2019) for the fungi, all containing the Illumina adapter sequences (see Supplementary Table S2 for an overview of the utilized primer sequences according to Hendgen et al., 2018). All PCRs were conducted using the proofreading KAPA Hifi polymerase (Kapa Biosystems, Boston, MA, United States). Each PCR reaction was carried out in a total volume of 15 μl containing 1 μl template DNA, 0.3 μl forward primer, 0.3 μl reverse primer, 7.5 μl 2x KAPA HiFi HotStar ReadyMix, and 5.9 μl nuclease free water; under the following thermocycling steps. 16S rDNA amplification: initial denaturation at 95°C for 3 min, followed by 25 cycles of denaturation at 98°C for 20 sec, annealing at 55°C for 15 sec, elongation at 72°C for 15 s, followed by a final extension at 72°C for 5 min. Fungal ITS2 amplification: initial denaturation at 95°C for 3 min, followed by 30 cycles of denaturation at 98°C for 20 s, annealing at 56°C for 20 s, elongation at 72°C for 20 s, followed by a final extension at 72°C for 5 min. Every sample was amplified in three replicates, resulting sample PCR products were checked by gel electrophoresis. The three replicates were pooled and cleaned-up using the Agencourt AMPure XP kit (Beckman Coulter, High Wycombe, United Kingdom). Subsequently, cleaned products were used as templates in an additional PCR, where Illumina Nextera XT indices and sequencing adaptors were attached according to the Illumina MiSeq protocol for amplicon preparation (Illumina Inc., San Diego, CA, United States). The amplifications followed these conditions: initial denaturation at 95°C for 3 min, 8 cycles of denaturation at 98°C for 30 s, annealing at 55°C for 30 s, followed by elongation at 72°C for 30 s, and a final extension at 72°C for 5 min. Resulting PCR products were purified again with AMPure beads. The libraries were then quantified by PicoGreen assays (Molecular Probes, Eugene, OR, United States) and pooled to provide equimolar representation. Fragment sizes and quality of DNA sequencing libraries were checked using an Agilent 2100 Bioanalyzer (Agilent Technologies, Palo Alto, CA, United States). The pool was used for paired-end sequencing of 2 × 300 bp with a MiSeq Reagent kit v3 on an Illumina MiSeq platform (Illumina Inc., San Diego, CA, United States) and was carried out at the Department of Soil Ecology of the Helmholtz-Centre for Environmental Research – UFZ in Halle (Saale), Germany.

### Bioinformatics Analysis

The raw reads were de-multiplexed by the Illumina MiSeq Reporter software package v2.5.1.3 with default settings. Retained fastq files without Illumina adaptors, sequencing primers and indices were analyzed using the pipeline DeltaMP (v0.2)^2^ by following the workflow presented in Schöps et al. (2018). In brief, soil-based Illumina sequences of 16S and ITS were processed and sequentially quality-filtered using mainly MOTHUR (v1.39.5-2, Schloss et al., 2009). Pair-end reads were merged with a minimum overlap of 20 bp using PandaSeq (v2.11, Masella et al., 2012). Sequences with any ambiguous base, more than four bp differences in the primer sequence, as well as homo-polymers with up to 20 bp differences were removed. Simultaneously, sequences, shorter than 50 or longer than 600 bp were discarded. Potential chimers were removed using UCHIME (Edgar et al., 2011) as implemented in MOTHUR (Schloss et al., 2009). Remaining sequences were pooled, de-replicated and sorted according to their abundance using OBITools (v1.2.11, Boyer et al., 2016). Unique sequences were clustered into operational taxonomic units (OTUs) with 97% sequence similarity using VSEARCH (v2.10.4, Rognes et al., 2016). By means of the Bayesian classifier as implemented in MOTHUR (Schloss et al., 2009), bacteria and fungi taxonomy was initially assigned using the SILVA reference database (v128, Quast et al., 2013) and UNITE (v8.0, Nilsson et al., 2018), respectively. The output was manually checked using Basic Local Alignment Search Tool (BLAST) of the National Center for Biotechnology (NCBI) (O’Leary et al., 2015). Plant derived 16S sequences assigned to chloroplasts or mitochondria were removed from the bacterial OTU table. Reads of samples were normalized at rarefaction depth of 96,167 and 26,578 reads per sample for bacteria and fungi, respectively, by using the function “rarefy_even_depth” from the phyloseq package 1.19.1 (McMurdie and Holmes, 2013) in R version 3.4.2 (R Development Core Team, 2017). The derived OTUs were assigned to their functional groups mainly based on FAPROTAX database (v1.1, Louca et al., 2016) and FUNGuild tool (v1.1, Nguyen et al., 2016) for bacteria and fungi, respectively. Raw sequences were deposited at the European Nucleotide Archive (ENA) and can be found under accession number PRJEB35688.

### Statistical Analyses

The statistical analyses were carried out using R, v3.4.2 (R Development Core Team, 2017). The microbial Shannon diversity index (Shannon, 1948) was calculated using the diversity function of the vegan package (Oksanen et al., 2017), and results were visualized via overlaid boxplots and stripcharts using the ggplot2 package (Wickham, 2016). We used a two-way analysis of variance (ANOVA) to compare the microbial diversity of PhytOakmeter root and root-free zones within and among the field sites. We then used Tukey HSD test to determine at which sites the tree root zone and root-free zone revealed significant difference (*p* < 0.05). In the same way, significant differences in microbial Shannon diversity among the sites’ tree root zones were analyzed. To explore how soil chemistry and weather parameters are correlated to the microbial Shannon diversity, multiple linear regression was done. We first removed auto-correlated parameters using the variance inflation factor (VIF < 5) (Akinwande et al., 2015), and the remaining parameters were differently combined into various models. The obtained regression models were then evaluated to choose the best approximating model by using Akaike’s Information Criterion (AIC) (Johnson and Omland, 2004). Subsequently, to determine whether the field sites contained significantly different microbial communities, the analysis of similarities (ANOSIM) permutation test (999 permutations) was used together with a non-metric multidimensional scaling (NMDS) based on the Bray-Curtis dissimilarity matrices (Clarke, 1993). We then applied the envfit function of the vegan package (Oksanen et al., 2017) to analyze correlation between structure of soil microbial communities and soil chemical parameters. Goodness-of-fit statistics (R2) were calculated based on 999 permutations. NMDS was also used to compare microbial community structure between PhytOakmeter root and root-free zones within respective sites, and ANOSIM was as well applied to test the statistical significance. Moreover, the overlap analysis of bacterial and fungal OTUs among different locations was done using the online tool venny (Oliveros, 2007/2015). Using DEseq2 (v1.24.0) via phyloseq (McMurdie and Holmes, 2013; Love et al., 2014), we distinguished which genera significantly increased presence in PhytOakmeter root zone over the tree root-free zone (*p* < 0.05). The results were then plotted using the graphical library ggplot2 (Wickham, 2016). By using all the OTUs found within the host tree root zone, we performed variance partitioning (varpart function in vegan) to assess the relative contribution of the environmental parameters and the host tree performance in explaining variation of the bacterial and fungal communities.

## Results

### Weather Data and Soil Chemical Parameters of the Field Sites

Details on weather data are summarized in Supplementary Table S1. The weather variables include precipitations as well as atmospheric and soil temperatures. There was no significant difference in any of the analyzed weather variables among the field sites.

The measured chemical parameters were mostly in similar ranges among the different field sites, even though some values differed significantly with, however, moderate difference amplitudes (Table 1). In particular, the soil of Greifenhagen and Bad Lauchstädt had lower values in SOC, hot and cold water extractable C and N.

The similarities among the soil parameters allowed repartition of the field sites into distinct groups. In this regard, concurrent similarity in pH and SOC grouped together Harsleben and Pfeiffhausen; C/N, HWC, and HWN put together Greifenhagen and Bad Lauchstädt; TN and C/N linked Bad Lauchstädt and Harsleben.

### PhytOakmeter Growth Performance Among the Field Sites

PhytOakmeter growth parameters within the respective field sites are summarized in Table 2. The PhytOakmeters outplanted in the four field sites had similar initial height. Also, among the field sites, there was no difference in percentage increase of the tree height during 2015 and 2016 vegetation periods. The number of shoot flushes produced by the trees during 2016 was comparable among the sites, but the first shoot flushes were significantly longer in Bad Lauchstädt than in the other sites.

**TABLE 2:** Tested growth parameters on the investigated PhytOakmeters within respective field sites.

Tree parameters	Harsleben	Pfeiffhausen	Greifenhagen	Bad Lauchstädt
Height at the outplanting time (cm)	65.8(±16.5)	71.7(±8.8)	78.8(±2.5)	75.3(±5.9)
Height percentage increase in 2015	26.5(±15.2)	34.7(±27.6)	31.9(±11.9)	64.4(±39.3)
Height percentage increase in 2016	32.6(±34.2)	17.8(±17.6)	33.2(±12.5)	38.1(±31.4)
Mean SF number in 2016	1.8(±0.4)	1.5(±0.5)	2.0(±0.0)	2.0(±0.9)
Mean first SF length in 2016	11.4(±10.7)^b^	10.0(±9.2)^b^	7.6(±4.4)^b^	27.3(±6.3)^a^
Leaf dry weight (g)	0.6(±0.3)	0.8(±0.4)	1.0(±0.3)	0.9(±0.3)
Ratio leaf dry weight to fresh weight	0.5(±0.1)^ab^	0.6(±0.1)^a^	0.5(±0.0)^a^	0.4(±0.0)^b^

*Values represent means of six selected trees (± standard deviation). Fresh and dry leaf weights represent total weights for five leaves of the main stem first shoot flush (SF1). Different superscript letters after standard deviations in a row mean statistically different (p < 0.05) according to a one-way ANOVA and Tukeys’ HSD test.*

### Overall Composition of Microbial Communities Among the Field Sites

For bacterial communities, a total of 5,092,013 reads representing 18,140 OTUs were obtained from the 38 samples from all four field sites. Removal of reads ascribed to chloroplasts and mitochondria gave a total of 5,066,965 reads corresponding to 17,890 OTUs, with a minimum of 96,167 and a maximum of 199,411 reads. Rarefaction to 96,167 reads per sample resulted in a total of 17,630 OTUs. For fungal community, the analysis availed a sum of 4,033 OTUs represented in a total of 1,545,424 reads; with minimum reads of 26,580 and a maximum of 56,794. Rarefaction to 26,578 reads per sample resulted in a sum of 3,970 OTUs. All rarefaction curves for both bacteria and fungi tended to approach the saturation plateau, an indication that the communities were almost exhaustively sampled and the data volume of sequenced reads was sufficient (see rarefaction curves in Supplementary Figure S3).

**FIGURE 1:**
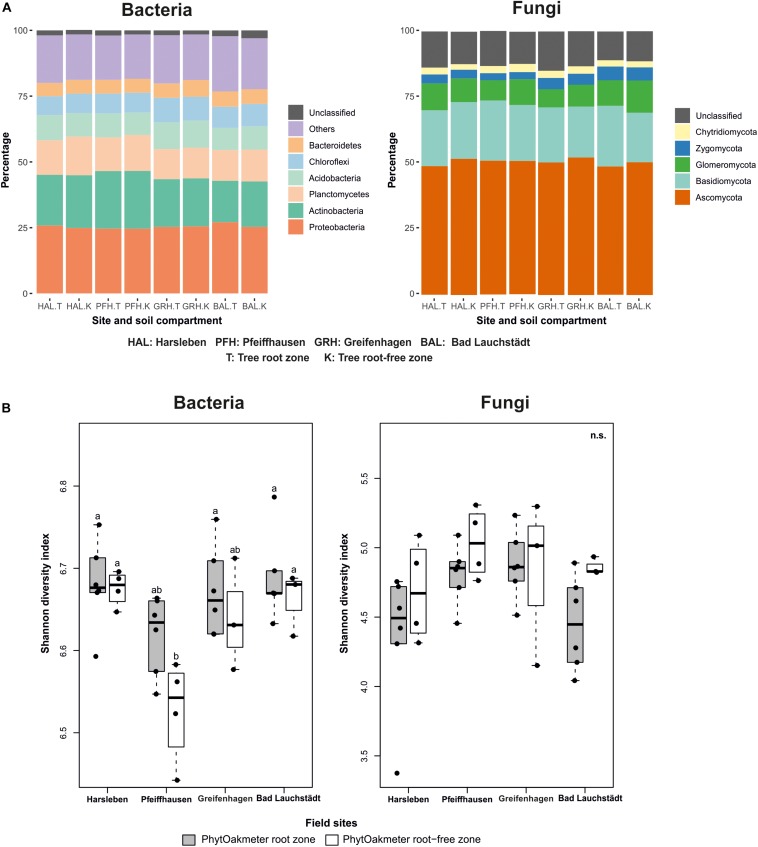
**(A)** Distribution overview of bacterial and fungal phyla between PhytOakmeter root and root-free zones within and among the field sites, **(B)** Shannon diversity index for bacteria and fungi within soils from PhytOakmeter root zone and the tree root-free zone of the respective field sites. Different letters above boxplots indicate significant differences (*p* < 0.05) according to Tukey-HSD *post hoc* test. n.s., not significantly different.

Overall, the rarefied bacterial OTUs were assigned to 42 different identifiable phyla, 126 classes, 169 orders, 319 families, and 582 genera. Bacterial communities were dominated by 13 phyla, with an individual relative abundance of at least 1%, all totaling up to 93% of the whole community. The five predominant phyla Proteobacteria, Actinobacteria, Planctomycetes, Acidobacteria and Chloroflexi covered more than 74% of the total community (Figure 1A). Unclassified OTUs at phylum level occupied 2.2%. All the bacteria phyla were similarly represented among all four field sites.

The rarefied fungal OTUs were classified into six different recognized phyla, 23 classes, 82 orders, 159 families, and 388 genera. The fungal phyla altogether were represented in the following order: Ascomycota (56.0%), Basidiomycota (26.2%), Glomeromycota (10.5%), former Zygomycota (4.0%), and Chytridiomycota (3.0%), with 14.6% unclassified. The fungal phyla were shared and also similarly represented among all the four field sites (Figure 1A).

### Microbial Shannon Diversity Associated With PhytOakmeter Root Zone, Field Sites and Environmental Parameters

Species diversity of both bacteria and fungi within PhytOakmeter root and root-free zones at each field site was determined by using the Shannon diversity index and results presented by boxplots (Figure 1B). The Shannon diversity values within the host tree root zones were similar among the sites for both bacteria and fungi. As well, species diversity of the host tree root-free zones was similar among the sites for both bacteria and fungi, except a significantly lower bacterial diversity value noticed at Pfeiffhausen. At each field site, the microbial species diversity values were also comparable between the host tree root and root-free zones. However, the species diversity of the host tree root zone tended to always be higher for the bacteria and, on the contrary, lower for the fungi.

As indicated by the lowest AIC values of the tested models (Supplementary Table S3), the best model to predict the microbial Shannon diversity included CWC, P, soil moisture and soil temperature for bacteria (*p* < 0.001 and adjusted *R*^2^ = 0.47), while it included CWC and soil temperature for fungi (*p* < 0.05, adjusted *R*^2^ = 0.12) (bold in Supplementary Table S3).

### Structure of Microbial Communities Among the Field Sites

**FIGURE 2:**
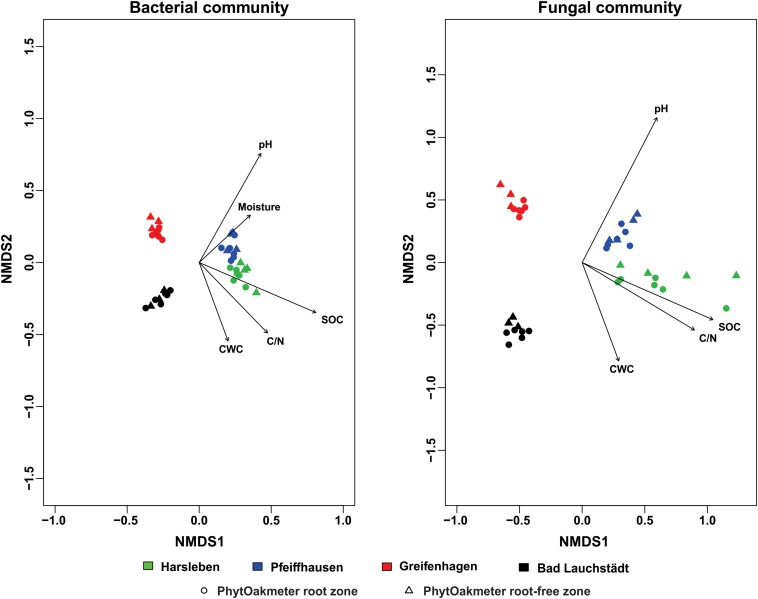
Non-metric multidimensional scaling (NMDS) based on Bray-Curtis dissimilarity displaying bacterial (stress = 0.07) and fungal (stress = 0.09) communities’ structure within field sites, and significantly correlated soil chemical parameters (*p* < 0.05).

ANOSIM showed that the structure of soil microbial communities was significantly site-specific for both bacteria (*p* < 0.001, *R* = 0.91) and fungi (*p* < 0.001, *R* = 0.82). This was visually supported by NMDS plots in which samples were ordinated in separate clusters according to the respective field sites (Figure 2). The NMDS plot displayed that the soil microbial communities of Harsleben and Pfeiffhausen were close to each other especially for bacteria (Figure 2). The figure also shows the significant impacts of soil pH, SOC, C/N, and CWC on the microbial community structure for both bacteria and fungi, plus soil moisture for only bacteria.

**FIGURE 3:**
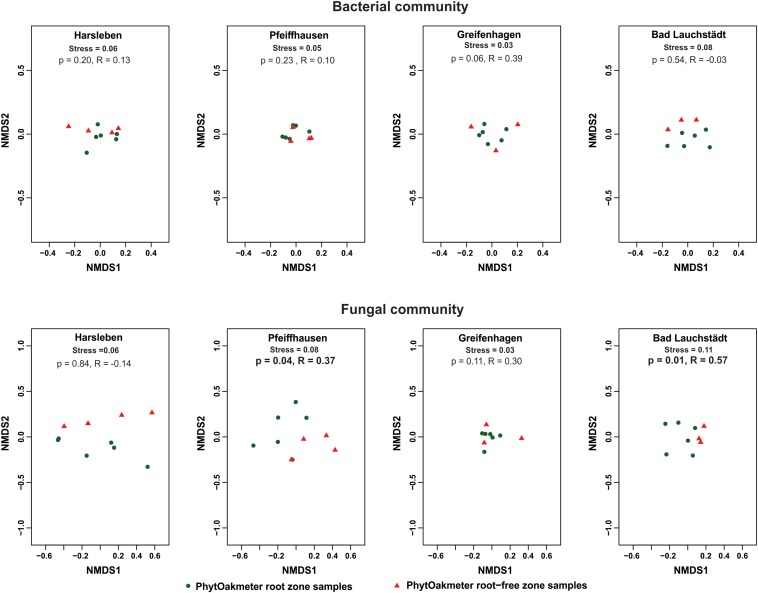
Non-metric multidimensional scaling (NMDS) based on Bray-Curtis dissimilarity displaying bacterial and fungal communities’ structure within respective field sites, and differentiating between the samples of PhytOakmeter root and root-free zones. p and statistic R values within respective sites are given by the analysis of similarities (ANOSIM) permutation test (999 permutations).

When we separately plotted samples of the respective sites, we visually found start of separation between microbial communities of PhytOakmeter root and root-free zones in one site (Bad Lauchstädt) for the bacteria and in three sites (Harsleben, Pfeiffhausen, and Bad Lauchstädt) for the fungi (Figure 3), indicating a beginning of the host tree effect on microbial community structure. However, ANOSIM only confirmed this host tree effect on fungal community in the field sites of Pfeiffhausen (*p* < 0.05, *R* = 0.37) and Bad Lauchstädt (*p* < 0.05, *R* = 0.57).

### Microbial Community Composition Within PhytOakmeter Root Zone in Comparison to the Tree Root-Free Zone

**FIGURE 4:**
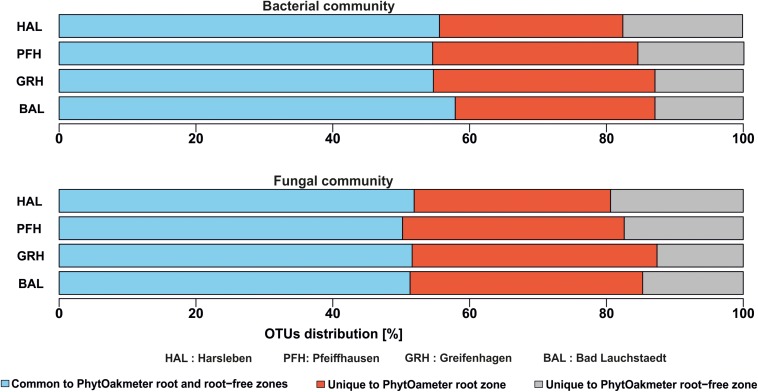
Overlap of bacterial and fungal OTUs between PhytOakmeter root zone and the tree root-free zone.

Composition of the soil microbial communities deduced from the OTUs overlap analysis between PhytOakmeter root zone and the tree root-free zone revealed significant differences (Figure 4). The highly abundant microbial OTUs tended to be generally shared between the two zones (55.7 and 51.2% for bacteria and fungi, respectively) while the least abundant tended to be uniquely detected within either zone. In this view, 29.6% bacterial OTUs and 32.7% fungal OTUs were exclusively detected within soil samples of the PhytOakmeter root zone, while 14.7% bacterial OTUs and 16.1% fungal OTUs were uniquely identified within the root free zone soil.

**FIGURE 5:**
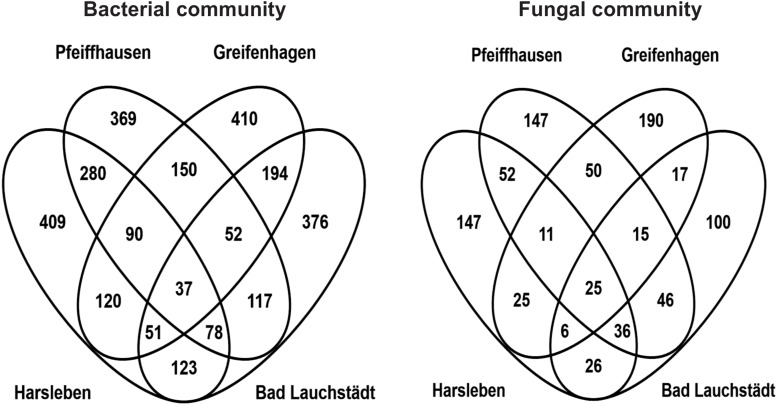
Venn diagrams showing an overlap of OTUs exclusive to PhytOakmeter root zone among the field sites.

Further overlap analysis separated the microbial OTUs exclusive to the tree root zone into those commonly found in all the field sites and those exclusive to either site (Figure 5). The common ones were considered as the putative “core microbiome” of the rooting zone of the DF159 clone. The detected core microbiome consisted of 37 and 25 OTUs for bacteria and fungi, respectively (Figure 5). The number of PhytOakmeter site-specific microbial OTUs ranged from 369 (Pfeiffhausen) to 410 (Greifenhagen) for bacteria, and from 100 (Bad Lauchstädt) to 190 (Greifenhagen) for fungi, and was always much higher than the number of the “core” OTUs.

**FIGURE 6:**
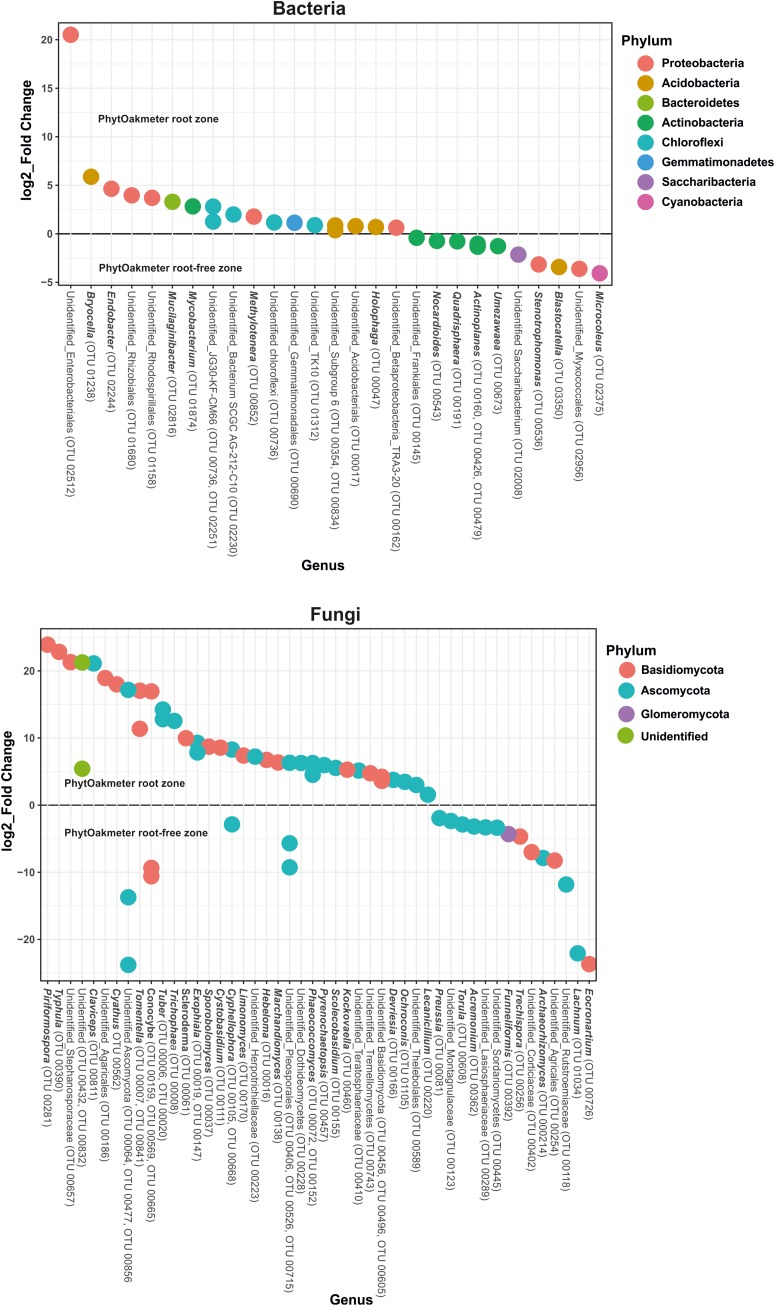
Differential abundance test for bacterial and fungal genera using Phyloseq and DESeq2. The graphs represents log_2__fold change of the microbial genera with significantly different abundance (*p* < 0.05) in the PhytOakmeter root zone compared to the tree root-free zone. A positive value signifies higher abundance while a negative value means lower abundance of the respective genera within the PhytOakmeter root zone compared to the tree root-free zone.

At the genus level, significant differences were also found between PhytOakmeter root and the tree root-free soil zones, as 27 bacterial and 48 fungal genera (including both the identified and unidentified) showed significant differential abundance between the two soil compartments (Figure 6, *p* < 0.05).

Specifically, Figure 6 shows, for bacteria, higher abundance of six identifiable genera and lower abundance of seven recognizable genera in the PhytOakmeter root zone compared to the tree root-free zone. The bacterial genera highly abundant within PhytOakmeter root zone in comparison to the tree root-free zone included *Bryocella*, *Endobacter*, *Mucilaginibacter, Mycobacterium, Methylotenara*, and *Holophaga*. Always in comparison to the tree root-free zone, we clearly noticed higher abundance of 23 identifiable fungal genera in the PhytOakmeter root zone. These consisted of, among others, *Piriformospora*, *Typhula*, *Claviceps*, *Cyathus*, *Tomentella*, *Tuber*, *Trichophaea*, *Scleroderma*, *Exophiala*, and *Hebeloma*. Eight recognizable fungal genera showed higher abundance in the tree root-free zone. To summarize, more differentially abundant genera were in the PhytOakmeter root zone compared to the tree root-free zone. Furthermore, among the highly abundant microbial genera within PhytOakmeter root zone, we noticed more fungal than bacterial genera.

### Compared Impacts of Soil Chemistry, Weather Parameters, and Host Tree Performance on Microbial Community Variation

**FIGURE 7:**
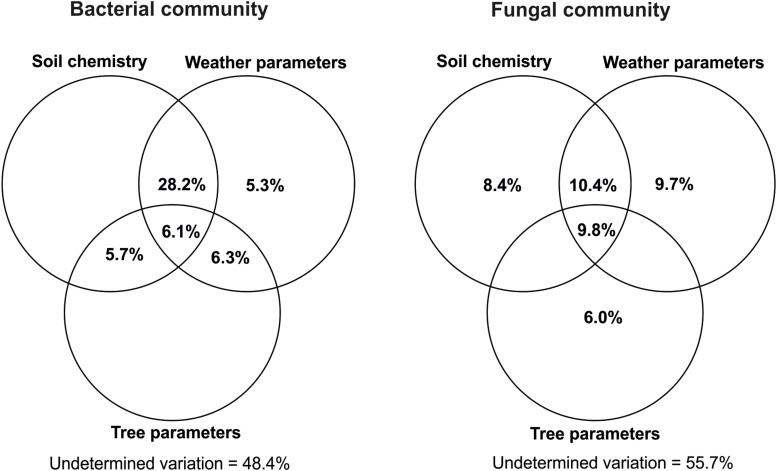
Variance partitioning analysis of the respective impacts of soil chemistry, weather, and host tree growth parameters on variations within bacterial and fungal communities. Soil chemistry included pH and soil organic matter content (SOC, TN, C/N, CWC, CWN, CWC/CWN, HWC, and HWN). Weather data included annual precipitations as well as monthly mean atmospheric and soil temperatures in the period of January 2014–September 2016. Tree growth-related parameters were height at the outplanting time, height increases in 2015 and 2016, shoot flushes produced in 2016 vegetative period, height of 2016 first shoot flush (SF1) as well as fresh and dry matter weight of SF1 leaves produced in 2016. Each circle represents the portion of variation accounted by each factor. Shared variance is represented by the intersecting portions of the circles. Values ≤ 0 are not shown. The calculations were done by using all the OTUs found within the host tree root zone.

Variance partitioning (Figure 7) showed that host tree performance traits alone could not explain any part of variation within the bacterial community while they accounted for 6.0% for the fungi. Similarly, the soil chemistry effect was only detectable for the fungi and explained 8.4%. Also, weather alone explained about 5.3% of the variance in bacteria and 9.7% in the fungi. The three types of factors had notably higher impacts when cumulating their single and combined effects derived from interactions with the other factors, whereby weather remained the strongest determinant followed by soil chemistry and, largely behind, tree performance. Even though this observation was similar in the two microbial groups, the explained variation was higher for bacteria than for fungi (Figure 7).

## Discussion

The current study revealed similar diversity levels of the microbiomes within PhytOakmeter root zone among the field sites and between the soil compartments (host tree root and root-free zones) within the individual sites. Our design was also adequate to detect specific changes in the community structure among the field sites. We also revealed different microbial composition between the PhytOakmeter root and root-free zones within respective sites. We were able to detect variations within the PhytOakmeter root zones amongst the sites and to separate the change fraction explained by the host tree from the one accounted for by the environmental parameters.

### Factors Equalizing the Microbial Diversity of PhytOakmeter Root and Root-Free Zones Within and Among the Field Sites

In our study, we partly confirmed our first hypothesis about microbial diversity levels in PhytOakmeter root zones among the field sites. However, we rejected the second hypothesis as we found no difference between the tree root and root-free zones within the individual sites. Despite small variations amongst the sites, this similar microbial diversity might mainly reflect comparable vegetation features and weather parameters among all the sites and between soil compartments (host tree root and root-free zones), which tended to equalize their microbiomes.

The first constant factor susceptible to homogenize the soil microbiomes of the field sites is the common genetic identity of the PhytOakmeters. As evidence to this PhytOakmeter clonal effect, microbial diversity within the tree root zones was similar among all the sites. Additionally and most importantly, bacterial diversity of the host tree root zone at Pfeiffhausen remained comparable to the tree root zones of the other sites in spite of its host tree root-free zone which was significantly different from most of its counterparts. According to previous reports, trees, especially through root exudates, provide specific carbon and energy sources to soil microorganisms. As a central source of nutrients, root exudates create therefore a niche for growth of microorganisms (Hassan et al., 2019), thus highly contributing to shaping the soil microbiome (Wieland et al., 2001; Garbeva et al., 2004; Nunan et al., 2005). Similar studies pointed out that variations in plant root exudates influence the diversity of the plant root microbiome (Grayston et al., 1998; El Zahar Haichar et al., 2008). As quantity and composition of root exudates are plant species-specific (Gransee and Wittenmayer, 2000; Gargallo-Garriga et al., 2018), each plant can shape its specific soil microbiome (Berg and Smalla, 2009). We can thus infer that genetically identical plants create within their root zones comparable microbial niches, resulting in similar diversity of their root-associated microbiomes.

Second, all the study sites share a similar climate with parallel weather variations. Temperature, the most important variable in defining the climate of a region, is one of the main factors influencing the occurrence, richness, stability, and activity of soil microorganisms (Borowik and Wyszkowska, 2016). Both atmospheric and soil temperatures were reported to impact on the soil microbiome (Alkorta et al., 2017). Atmospheric temperature has direct effect on soil temperature and indirectly affects host plant productivity as well as availability of carbon sources for microbial growth (Anderson, 1992; Bardgett et al., 1999). Also, both directly and indirectly, soil temperature significantly shapes the conditions for growth and development of microorganisms (Borowik and Wyszkowska, 2016). Directly, soil temperature influences microbial metabolism while the indirect effects are noticed via its impacts on plant productivity (Jefferies et al., 2010). Comparable atmospheric and soil temperatures amongst the study field sites may have also had an important contribution to the similar microbial diversity.

Lastly, all the sites are grassland. As roots of herbaceous plants highly impact soil microbial communities (Burke et al., 2009), herbaceous plant cover may have contributed a lot to the noticed similar microbial Shannon diversity between host tree root and root-free zones within individual sites. This assumption is supported by Christie et al. (1978) who reported that one plant root-associated microbiome can be influenced by neighboring plants. Therefore, herbaceous plant cover may have extended their effect to the PhytOakmeter root zone and, thus, contributed to homogenize microbial diversity between the host tree root and root-free zones at the individual grassland field sites.

### Differences in Microbial Community Structure Among the Field Sites

As indicated by NMDS plots and ANOSIM, structure of the microbial communities was in fact revealed different from site to site in spite of their similar microbial diversity levels. With this, we rejected the second part of our first hypothesis which predicts high similarity in microbial community structure among the field sites. In general, the noticed difference might reflect the micro-heterogeneity of soil habitat (Buscot, 2005) among the sites in addition to their land use history. Besides, spatial isolation among the field sites may have also contributed to their differences in microbial community structure. According to various reports, spatial isolation leads to microbial species endemic to specific field sites (Zhou et al., 2002) and, therefore, to variations in soil microbial community, even within a single region (Bokulich et al., 2014; Zarraonaindia et al., 2015; Gourmelon et al., 2016).

Differences in soil pH and organic matter content can also be used to further explain the different microbial communities among the sites. This is supported by previous reports such as Eiland et al. (2001), Fierer and Jackson (2006), Medeiros et al. (2006), Zhalnina et al. (2015), and Xue et al. (2018). From this view, repartition of the sites into distinct groups, as shown by our NMDS plot analyses, can be explained relying on similarities in soil pH and organic matter content. Comparable pH, SOC, and C/N between Harsleben and Pfeiffhausen matched with the NMDS plot results where their soil microbiomes were found to be more similar. Comparable C/N and TN content between Bad Lauchstädt and Harsleben are also consistent with the similarity level of their respective microbial communities. In the same way, similar level of C/N, HWC, and HWN between Greifenhagen and Bad Lauchstädt relate to their comparable microbial community structure.

On the contrary, all the sites had the same microbial phyla with similar proportion. Proteobacteria and Ascomycota dominated the overall bacterial and fungal communities, respectively. High abundance of Proteobacteria was previously reported within numerous types of ecosystems, such as in grasslands (Singh et al., 2007), croplands (Tian and Gao, 2014), forest-grass ecosystems (Zeng et al., 2016), and natural hardwood forest soils (Lin et al., 2011). Ascomycota were reported dominant in soil fungal communities of semi-arid (Porras-Alfaro et al., 2011) and temperate (Prober et al., 2015; Chen et al., 2017) grasslands, oppositely to forest soils dominated by Basidiomycota (Goldmann et al., 2015; Terhonen et al., 2019).

### Differences in Microbial Community Composition Between Soils of PhytOakmeter Root and the Root-Free Zones

Comparison between the PhytOakmeter root and root-free soil compartments confirmed our third initial hypothesis about higher abundance of some particular soil microbial taxa in the PhytOakmeter root zone. We found more microbial OTUs exclusive to the host tree root zone than the OTUs uniquely detected within the tree root-free zone. This indicates that, after two years of their field outplant, PhytOakmeter trees had already exerted significant effect on local microbial communities regardless of legacy effects of previously existing vegetation. This opposes Elgersma et al. (2011) who reported soil microbial structure to be not affected by the current vegetation two years after transplantation, rather largely determined by the legacy effect of the previous vegetation type. Examination of the PhytOakmeter root-associated microbial OTUs showed a PhytOakmeter “core” microbiome as well as a PhytOakmeter “site-specific” microbiome. Following the definition by Shade and Handelsman (2012), the PhytOakmeter “core” microbiome referred to bacterial and fungal OTUs exclusively found within the tree root zone in all the sites. Such a core microbiome has been estimated to likely play a key role in the plant soil systems among variable sites (Shade and Handelsman, 2012; Shakya et al., 2013). In the current study, however, all the PhytOakmeter “core” microbial OTUs were not identified for specific functions to the host tree itself, neither to the whole ecosystem. We also revealed PhytOakmeter site-specific microbial species, and this supported the view that plants recruit root-associated microorganisms from surrounding soil microbial reservoirs (Compant et al., 2019). The microbial recruitment by host plant roots was reported to depend on composition of the local microbial pool and microbial-host plant affinities designated as microbial host fidelity and preference (Bonito et al., 2014; Compant et al., 2019). In herbaceous plants, this process was shown to be promoted by nutrients and signaling molecules present in the plant exudates (Marschner et al., 2004; Prescott and Grayston, 2013; Jacoby et al., 2017). Similar processes were also observed for trees (Landeweert et al., 2001; Gahan and Schmalenberger, 2014). Metabolites exuded by the host tree serve to recruit and subsequently support or inhibit multiplication of particular microbial taxa within the tree root zone (Garbeva et al., 2004; Bais et al., 2006; Lareen et al., 2016). In line with these previous findings, our current study also revealed some highly abundant bacterial and fungal genera in the PhytOakmeter root zone compared to the tree root-free zone of soils.

Plant roots can attract beneficial microorganisms from surrounding soil, and those play important roles in plant performance especially by improving plant mineral nutrition. Even though there is still limited knowledge on which particular microbes are good partners for boosting plant nutrition, it has been postulated that plants have evolved specific recognition mechanisms to discriminate beneficial microorganisms from those that need to be repelled (Jacoby et al., 2017). In the current study, none of the differentially abundant bacterial genera between PhytOakmeter root and root-free zones could be identified for their potential function. Contrarily, we were able to annotate ecological functions to a certain number of the highly abundant fungal genera within the PhytOakmeter root zone. They included *Tomentella*, *Tuber*, *Trichophaea*, *Scleroderma*, *Exophiala*, and *Hebeloma* which are ectomycorrhizal (Tedersoo et al., 2010). The ectomycorrhizal fungi assist their associated plants to draw more nutrients and water from the soil as well as to increase the plant tolerance to different environmental stresses (Tedersoo et al., 2010). In recruiting the ectomycorrhizal fungal genera, the PhytOakmeter trees may have been targeting such an important contribution to the host plant health. Compared to the tree root-free zone, PhytOakmeter root zone was also enriched in yeast genera *Phaeococcomyces* (Butler et al., 2004), *Sporobolomyces* (Wang et al., 2015), *Cystobasidium* (Ramos-Garza et al., 2015; Yurkov et al., 2015), and *Cyphellophora* (Feng et al., 2014). Yeasts are essential in ecological processes involving mineralization of organic matter (Botha, 2011). The tree root zone incorporated as well *Marchandiomyces* whose several species are lignicolous (DePriest et al., 2005; Lawrey et al., 2008), and saprotrophic genera such as *Ochroconis* (Gams, 2015) and *Typhula* (Shiryaev and Kotiranta, 2007) which participate in breaking down of complex organic molecules. Our findings agree with the previously reported ectomycorrhizal status of oaks (Herrmann and Buscot, 2007) and the tree ability to interact with large microbial communities which assist in nutrients acquisition (Jumpponen and Jones, 2009; Tarkka et al., 2013). The tree root-associated microorganisms are well-known to serve in improving tree health and nutrition, preventing establishment of pathogens, and adapting to specific local environmental conditions (Uroz et al., 2016; Gehring et al., 2017; Lau et al., 2017).

### Microbial Communities in the Host Tree Root Zone Are Shaped More by Environmental Parameters Than by the Host

Contribution of the environmental parameters to variations within bacterial and fungal communities of the PhytOakmerer root zone soil was found to be higher than contribution of the tree growth-related parameters. This finding might be due to two main reasons: (1) Host trees were still very young (only two years, i.e., two vegetation periods, in the field). Even more, the first vegetation period for trees after field release corresponds to a transplant shock. This period consists of acclimation to local soil environment and regeneration of the root system (Hargrave et al., 2002). After the transplant shock period, PhytOakmeters had practically only one single vegetation period to impact on surroundings and, apparently, this was not enough to exert a huge effect on local soil microbial community. The dependency of soil microbial community on the host tree age seems to be high. As previously proved, soil microbial communities associated with roots of perennial plant change in both richness and composition over the host’s lifetime. After out-planting, the plants replace a common soil microbial community they were exposed to as saplings with local communities of their respective field sites. From there on, the host plants continue to shape their respective root-associated microbial communities. These development dynamics were previously reported by Wagner et al. (2016) and Goldmann et al. (2020). (2) The soil was sampled in the tree root zone rather than rhizosphere where high tree effect on microbial community could be expected. As previously reported, the rhizosphere is known as a nutrient-rich compartment in the soil influenced by the plant. In the rhizosphere, carbon compounds, which serve as the main food and energy source for soil microbes, are continuously introduced via rhizodeposition and sloughed-off cells (Breidenbach et al., 2016). Sampling the host PhytOakmeter root zone rather than the rhizosphere led to dilute the host tree influence on the soil inhabiting microorganisms. However, even though tiny, the impact revealed at this young age of the trees is remarkable especially in the context of a temperate climate that does not promote rapid tree growth. Until now such quick effects of tree planting on soil microbial communities had been reported in the subtropics (Weißbecker et al., 2018).

## Conclusion and Future Perspectives

In conclusion, there is a high similarity in microbial biodiversity among the field sites but their microbial community structure is different. Even though still young, the capability of PhytOakmeters to recruit a specific beneficial microbiome in their root zone from surrounding microbial reservoirs was evidenced. The study revealed concurrent impact of environmental parameters and the host PhytOakmeter in shaping soil microbiome of the host tree root zone, but the magnitude of environmental parameters was higher than the impact of the host tree. Since this finding is likely based on the age of the trees, a similar study with older host trees is needed. For this, further measures of soil properties, such as information on texture, might even explain more microbial variance. Ideally, the investigation of the root endophytic compartment and/or the rhizosphere would be beneficial to unravel the PhytOakmeter-microbe interaction further. Moreover, the analysis of PhytOakmeter effects on soil microbiome at a large-scale is also required to move toward a comprehensive understanding of the tree root microbiome assemblage, and to have a better overview on mutual impacts between host tree and environmental variables in shaping the tree root zone microbiome. Nevertheless, our presented approach is an important step toward more integrative studies using clonal trees, and provides an opportunity to perform long-term interaction biomonitoring.

## Data Availability Statement

The raw sequences generated for this study can be found in the European Nucleotide Archive (ENA). Accession number PRJEB35688.

## Author Contributions

FB and SH conceived the project and designed the sampling. SH performed the sampling. JH, SH, and TR performed the laboratory works. JH and KG analyzed and interpreted the results. JH and KG wrote the manuscript with input from SH, TR, and FB. All authors contributed to revisions of the manuscript.

## Conflict of Interest

The authors declare that the research was conducted in the absence of any commercial or financial relationships that could be construed as a potential conflict of interest.

## Footnotes

1 www.tereno.net

2 https://github.com/lentendu/DeltaMP/
