# Supplementary material for: Tree Root Zone Microbiome: Exploring the Magnitude of Environmental Conditions and Host Tree Impact
Source: Front Microbiol. 2020 Apr 23;11:749. doi: 10.3389/fmicb.2020.00749 (PMC7190799; doi:10.3389/fmicb.2020.00749)
Supplement: Supplementary file 1 [file Data_Sheet_1.docx]

**Tree root zone microbiome: exploring the magnitude of environmental conditions and host tree impact**

Jean de Dieu Habiyaremye, Kezia Goldmann, Thomas Reitz, Sylvie Herrmann, François Buscot

**Supplementary information**

**Supplementary figures**

**
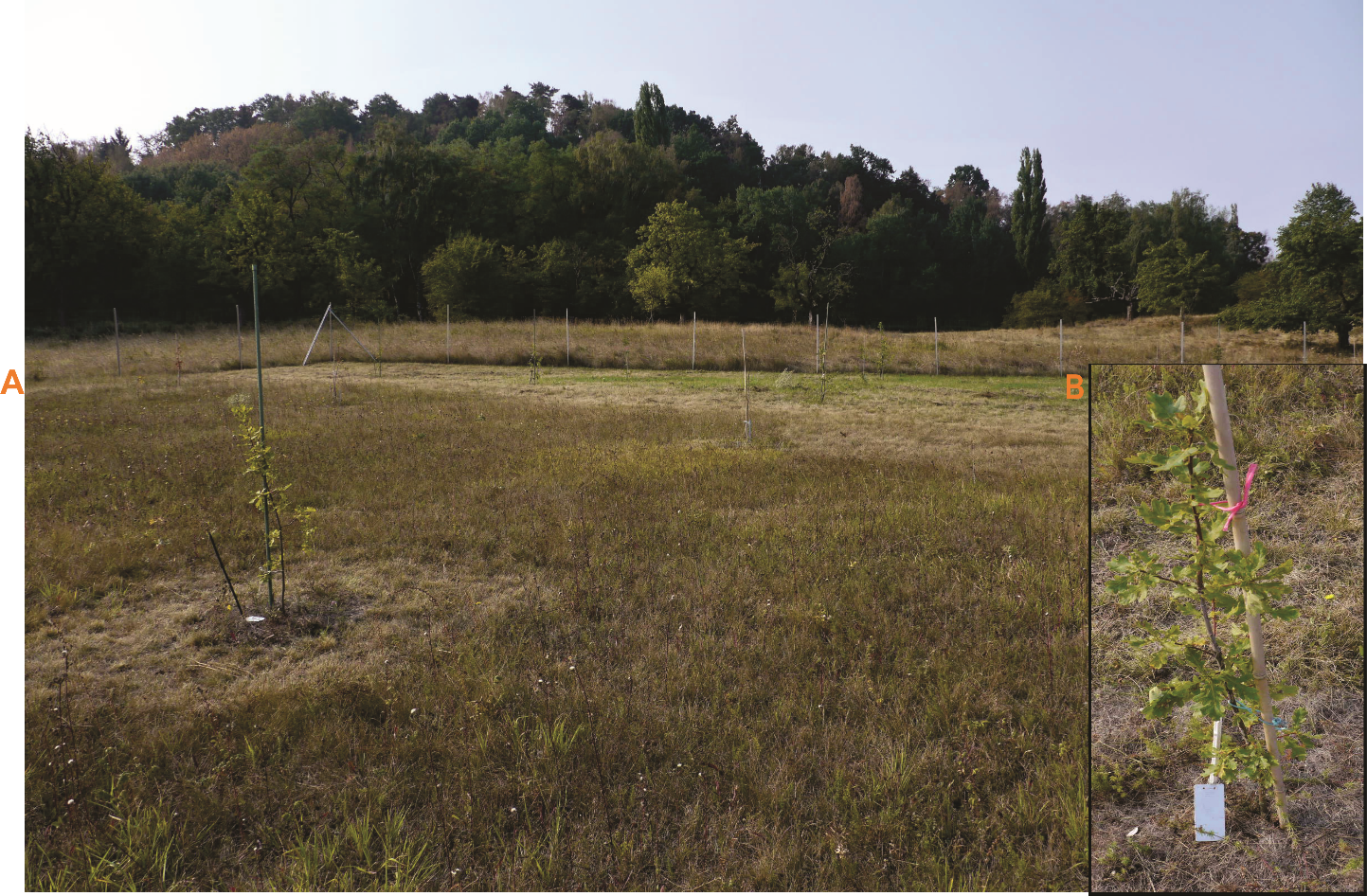
**

**Supplementary Figure S1.** Harsleben field site at sampling time in November 2016. Harsleben and the other field sites are grasslands with plot soil surface entirely covered by herbaceous plants. **(A)** General overview of the field plot. (**B**) Zoomed-in PhytOakmeter tree within the field plot.

**
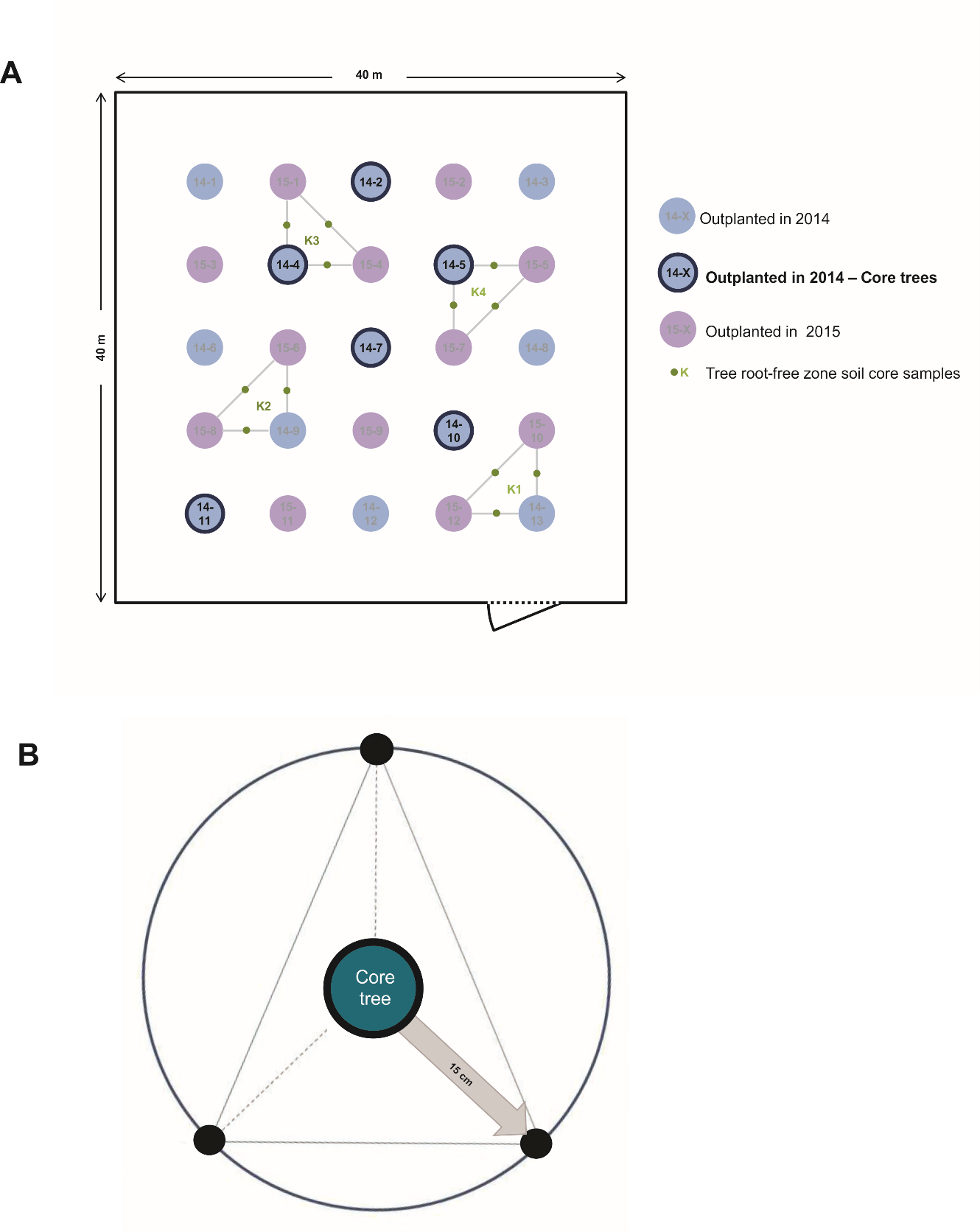
**

**Supplementary Figure S2. (A)** Plot sampling design overview, case of Harsleben field site. In total, ten samples were taken (six samples were taken within root zone of six core trees and four samples were taken in the tree root-free zone). The three subsamples of each tree root-free zone were taken in positions illustrated by green dots, and pooled to respectively make composite samples K1, K2, K3 and K4. **(B)** Sampling positions within PhytOakmeter root zone. As indicated by three black balls, the three subsamples of every tree root zone were taken at 120° angle around a selected tree, 15 cm horizontal distance from the tree trunk, and pooled together.

**
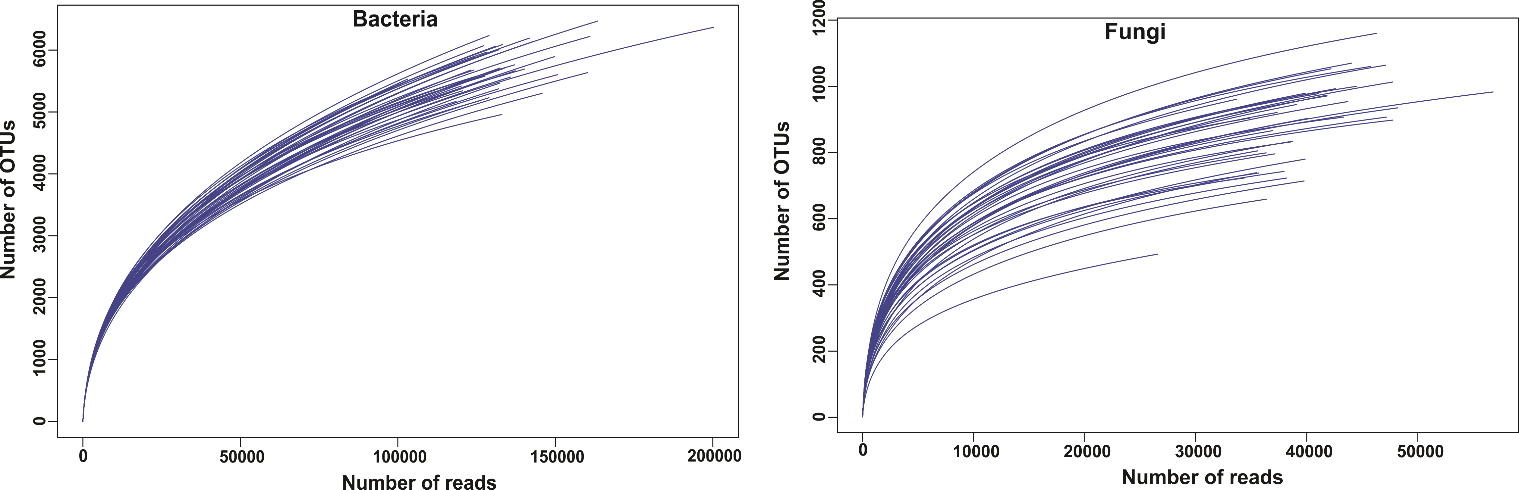
**

**Supplementary Figure S3.** Individual rarefaction curves of bacterial and fungal OTUs at a 97% similarity level of all 38 soil samples.

**Supplementary tables**

**Supplementary Table S1.** Weather data among the study field sites. The parameters were measured at the weather stations of the Helmholtz Centre for Environmental Research-UFZ. Atmospheric temperatures were measured at 200 cm above soil surface, while soil temperatures represent an average of the upper 20 cm calculated from single measurements at 5, 10, and 20 cm soil depth. The here presented weather data encompass the period of January 2014 (year of host trees out-planting) to December 2016, but the ones used for further analysis go up to September 2016 (time of soil sampling). According to one-way ANOVA, no significant differences were observed amongst the sites’ mean values.

| Weather variables | Parameter | Harsleben | Pfeiffhausen | Greifenhagen | Bad Lauchstädt |
| --- | --- | --- | --- | --- | --- |
| Precipitation [mm] | Annual total 2014 | 555.2 | 540.5 | 612.4 | 452.6 |
|  | Annual total 2015 | 516.9 | 439.6 | 488.2 | 399.9 |
|  | Annual total 2016 | 365.9 | 333.2 | 407.4 | 437.2 |
|  | January-September 2016 | 274.8 | 248.8 | 298.5 | 337.1 |
|  | Grand total 2014-Sep 2016 | 1,346.9 | 1,228.9 | 1,399.1 | 1,189.6 |
|  |  |  |  |  |  |
| Atmospheric temperature [°C] | Annual mean 2014(+SD) | 11.1 (±5.9) | 10.8 (±6.2) | 10.1 (±6.0) | 11.0 (±6.2) |
|  | Annual mean 2015(+SD) | 10.6 (±6.1) | 10.3 (±6.2) | 9.7 (±6.3) | 10.7 (±6.5) |
|  | Annual mean 2016(+SD) | 12.3 (±7.2) | 11.9 (±7.6) | 11.0 (±7.5) | 12.1 (±7.6) |
|  | Overall mean (+SD) | 11.2 (±6.2) | 10.9 (±6.4) | 10.2 (±6.3) | 11.2 (±6.5) |
|  | Maximum monthly mean | 20.7 | 20.6 | 20.2 | 21.2 |
|  | Minimum monthly mean | 1.7 | 0.5 | 0.2 | 1.0 |
|  |  |  |  |  |  |
| Soil temperature [°C] | Annual mean 2014(+SD) | 11.6 (±6.2) | 11.4 (±6.5) | 10.7 (±5.7) | 11.9 (±6.9) |
|  | Annual mean 2015(+SD) | 11.1 (±6.5) | 10.6 (±6.7) | 10.0 (±6.0) | 11.4 (±7.3) |
|  | Annual mean 2016(+SD) | 13.0 (±7.4) | 12.3 (±7.5) | 11.7 (±6.9) | 13.1 (±8.0) |
|  | Overall mean (+SD) | 11.9 (±6.7) | 11.4 (±6.9) | 10.7 (±6.2) | 12.1 (±7.2) |
|  | Maximum monthly mean | 21.3 | 20.9 | 19.5 | 22.3 |
|  | Minimum monthly mean | 2.0 | 1.3 | 1.6 | 1.7 |

Data source*: Meteorological data, Helmholtz Centre for Environmental Research – UFZ*

**Supplementary Table S2.** Overview of the used bacterial 16S rDNA and fungal ITS2 primers (Hendgen et al., 2018). Abbreviations according to IUPAC Ambiguity Code: A - adenine, C - cytosine, G - guanine, T -tyrosine, N - “aNy” base, ie A, C, G or T/U (Johnson, 2010)

| Primer name | Primer sequence 5’-3’ |
| --- | --- |
| P5-8N-515F | ACACTCTTTCCCTACACGACGCTCTTCCGATCTNNNNNNNNGTGCCAGCMGCCGCGGTAA |
| P5-7N-515F | ACACTCTTTCCCTACACGACGCTCTTCCGATCTNNNNNNNGTGCCAGCMGCCGCGGTAA |
| P7-2N-806R | GTGACTGGAGTTCAGACGTGTGCTCTTCCGATCTNNGGACTACHVGGGTWTCTAAT |
| P7-1N-806R | GTGACTGGAGTTCAGACGTGTGCTCTTCCGATCTNGGACTACHVGGGTWTCTAAT |
| P5-5N-ITS4 | ACACTCTTTCCCTACACGACGCTCTTCCGATCTNNNNNTCCTCCGCTTATTGATATGC |
| P7-4N-fITS7 | GTGACTGGAGTTCAGACGTGTGCTCTTCCGATCTNNNNGTGARTCATCGAATCTTTG |

**Supplementary Table S3**: Results of linear model analysis testing the correlation between environmental parameters and the microbial Shannon diversity index results. We first removed auto-correlated parameters using the variance inflation factor (VIF < 5); then the remaining parameters were differently combined into various models and tested against the microbial Shannon diversity index results. The obtained regression models were then evaluated to choose the best approximating model by using Akaike’s Information Criterion (AIC). Based on AIC values and significant correlation to the microbial Shannon diversity, the best model included CWC, P, soil moisture and soil temperature for bacteria (p < 0.001 and adjusted R^2^ = 0.47), while it included CWC and soil temperature for fungi (p < 0.05, adjusted R^2^ = 0.12).

|  | Bacteria | | | Fungi | | |
| --- | --- | --- | --- | --- | --- | --- |
| Model parameters/components | AIC | p | Adjusted R^2^ | AIC | p | Adjusted R^2^ |
| pH, TN, C/N, CWC, NO_3_-N, total mineral N, K, P, soil moisture and soil temperature | -93.0 | <0.01 | 0.44 | 53.6 | 0.08 | 0.22 |
| TN, C/N, CWC, K, P, soil moisture and soil temperature | -105.1 | <0.001 | 0.49 | 50.3 | 0.21 | 0.09 |
| CWC, K, P, soil moisture and soil temperature | -111.9 | <0.001 | 0.49 | 42.9 | 0.12 | 0.11 |
| **CWC, P, soil moisture and soil temperature** | **-112.6** | **<0.001** | **0.47** | 41.9 | 0.13 | 0.09 |
| CWC, soil moisture and soil temperature | -111.3 | <0.001 | 0.43 | 39.5 | 0.08 | 0.11 |
| **CWC and soil temperature** | -111.9 | <0.001 | 0.41 | **37.4** | **0.04** | **0.12** |
| CWC, P and soil temperature | -112.9 | <0.001 | 0.45 | 39.5 | 0.08 | 0.11 |

References

Hendgen, M., Hoppe, B., Döring, J., Friedel, M., Kauer, R., Frisch, M., Dahl, A., and Kellner, H. (2018). Effects of different management regimes on microbial biodiversity in vineyard soils. *Scientific Reports* 8**,** 9393. doi: 10.1038/s41598-018-27743-0

Johnson, A.D. (2010). An extended IUPAC nomenclature code for polymorphic nucleic acids. *Bioinformatics (Oxford, England)* 26**,** 1386-1389. doi: 10.1093/bioinformatics/btq098
